# Understanding the effect of oxide components on proton mobility in phosphate glasses using a statical analysis approach†

**DOI:** 10.1039/d0ra10327f

**Published:** 2021-01-14

**Authors:** Takahisa Omata, Issei Suzuki, Aman Sharma, Tomohiro Ishiyama, Junji Nishii, Toshiharu Yamashita, Hiroshi Kawazoe

**Affiliations:** Institute of Multidisciplinary Research for Advanced Materials, Tohoku University Katahira 2-1-1 Sendai 980-8577 Japan takahisa.omata.c2@tohoku.ac.jp +81-22-217-5832 +81-22-217-5832; Fuel Cell Materials Group, Research Institute for Energy Conservation, National Institute of Advanced Industrial Science and Technology (AIST) AIST Central 5, Higashi 1-1-1 Tsukuba Ibaraki 305-8565 Japan; Research Institute for Electronic Science, Hokkaido University Kita 21 Nishi 10, Kita-ku Sapporo 001-0021 Japan; Kawazoe Frontier Technologies Corporation Kuden-cho 931-113, Sakae-ku Yokohama 247-0014 Japan

## Abstract

The models to describe the proton mobility (*μ*_H_) together with the glass transition temperature (*T*_g_) of proton conducting phosphate glasses employing the glass composition as descriptors have been developed using a statical analysis approach. According to the models, the effects of additional HO_1/2_, MgO, BaO, LaO_3/2_, WO_3_, NbO_5/2_, BO_3/2_ and GeO_2_ as alternative to PO_5/2_ were found as following. *μ*_H_ at *T*_g_ is determined first by concentrations of HO_1/2_ and PO_5/2_, and *μ*_H_ at *T*_g_ increases with increasing HO_1/2_ concentration and decreasing PO_5/2_. The component oxides are categorized into three groups according to the effects on *μ*_H_ at *T*_g_ and *T*_g_. The group 1 oxides increase *μ*_H_ at *T*_g_ and decrease *T*_g_, and HO_1/2_, MgO, BaO and LaO_3/2_ and BO_3/2_ are involved in this group. The group 2 oxides increase both *μ*_H_ at *T*_g_ and *T*_g_, and WO_3_ and GeO_2_ are involved in this group. The group 3 oxides increase *T*_g_ but do not vary *μ*_H_ at *T*_g_. Only NbO_5/2_ falls into the group 3 among the oxides examined in this study. The origin of the effect of respective oxide groups on *μ*_H_ at *T*_g_ and *T*_g_ were discussed.

## Abstract 2

Relationship between composition and proton mobility in proton conducting phosphate glass has been obtained using a statical analysis approach.

## Graphical abstract

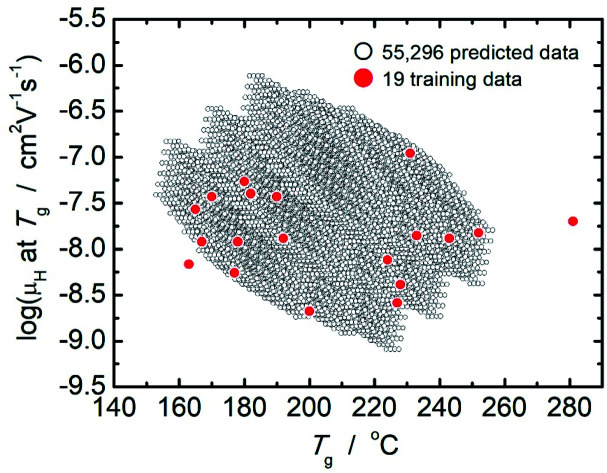

## Introduction

Inorganic glasses have been studied for decades as solid electrolytes because of their electrochemical stability and chemical durability, and various cationic conduction, such as Li^+^, Ag^+^ and H^+^ conduction, in oxide glass has been investigated extensively.^1–3^ Recent demands for highly proton conducting electrolytes in the temperature range between 250 and 500 °C that is operating temperatures of intermediate temperature fuel cells accelerate to explore proton conducting glasses.^4–9^ Our group developed a technique termed as alkali-proton substitution (APS) that injects high concentration of proton carriers, >10^21^ cm^−3^, into phosphate glasses^10^ and fabricated many proton conducting glasses by using APS.^11–14^ We studied characteristics of glasses that influence on proton conductivity, such as polymerization level of phosphate framework (ratio of the number of oxygen to phosphorous atoms; O/P ratio)^15^ and kinds of glass network modifier,^16^ and the effect of additional glass-network formers, such as GeO_2_, on the thermal stability.^17^ As a result, 2 × 10^−3^ S cm^−1^ of proton conductivity at 300 °C has been achieved by 34HO_1/2_–2NaO_1/2_–4NbO_5/2_–2BaO–4LaO_3/2_–4GeO_2_–1BO_3/2_–49PO_5/2_ glass (36H-glass) up to now.^18^ Based on the electromotive force and electrochemical hydrogen pump experiments, the phosphate glass electrolyte is confirmed that the mean transport number of proton is unity even under the oxidation atmosphere like an air electrode atmosphere in the fuel cell,^19^ suggesting that highly efficient operation of fuel cells and steam electrolysis cells is achievable owing to its no electronic leakage.^20^ In addition, fabrication of ultra-thin glass electrolytes with a thickness of 16 μm was recently demonstrated by the press forming.^21^ This will be a great advantage of the glass electrolyte in order to reduce electrolyte resistance (ohmic resistance) of the electrochemical cells. However, further increase of their proton conductivity >1 × 10^−2^ S cm^−1^ at the operating temperature is still required for practical applications.

Very recently, we have found that the mobility of proton carriers (*μ*_H_) at the glass transition temperature (*T*_g_) in phosphate glasses converges in a small range between 2 × 10^−9^ and 2 × 10^−7^ cm^2^ V^−1^ s^−1^, whereas *T*_g_ of the glasses is in the wide range of 150 to 650 °C, proton conductivity at 200 °C is also wide range of 10^−10^ to 10^−4^ S cm^−1^, and proton carrier concentration is in the range of 10^19^ to 10^22^ cm^−3^.^22^ Because the *μ*_H_ at *T*_g_ of the 36H-glass is 5.4 × 10^−8^ cm^2^ V^−1^ s^−1^ that is the middle in the *μ*_H_ at *T*_g_ range from 2 × 10^−9^ to 2 × 10^−7^ cm^2^ V^−1^ s^−1^, it is suggested that its proton conductivity can be further increased by improving the *μ*_H_ at *T*_g_. Because the determining factor of *μ*_H_ at *T*_g_ of proton conducting phosphate glasses has yet to be cleared, we unfortunately still do not understand how to improve *μ*_H_ at *T*_g_.

The composition of glasses is continuatively controllable unlike the crystalline materials; therefore, various properties of glasses, have been empirically expressed by the mole fraction weighting mean of the respective components.^23–26^ Whereas to understand the effects of fundamental properties of glasses, such as O–H bonding, local structure surrounding protons and short range atomic structure of the glass framework, on *μ*_H_ at *T*_g_ are of course important to understand the proton conduction in phosphate glasses from the physical aspect, understanding the relationship between the glass composition and *μ*_H_ at *T*_g_ is also valuable in order to improve the electrolyte performance of proton conducting phosphate glasses. When the proton conductivity is successfully described by the glass composition, the proton conductivity of phosphate glasses will be easy to improve based on the obtained relationship between the glass composition and *μ*_H_ at *T*_g_, and that will have a major impact on the electrochemical cells such as fuel cells and steam electrolysis cells working at intermediate temperatures. The proton conducting phosphate glasses prepared by using APS previously reported consists of many oxide components;^22^ for example, 36H-glass involves 8 oxides as HO_1/2_, NaO_1/2_, BaO, LaO_3/2_, NbO_5/2_, GeO_2_, BO_3/2_ and PO_5/2_; therefore, it is not easy to understand the role of the respective component oxides on *μ*_H_ at *T*_g_ and the relationship between the composition and *μ*_H_ at *T*_g_.

Here, we have developed a model, using a statical analysis approach, to describe *μ*_H_ at *T*_g_ of phosphate glasses according to the glass composition, *i.e.*, the mol% of respective component oxides were employed as descriptors. We also developed a model to describe *T*_g_ because the thermal stability of proton conducting glasses is another key property taking the working temperature of the electrochemical devices involving the glasses into account. The effect of respective component oxides on *μ*_H_ at *T*_g_ and *T*_g_ were discussed based on the model obtained.

## Methodology

### Dataset details

The dataset for *μ*_H_ at *T*_g_ and *T*_g_ of proton conducting phosphate glasses used as training data in this study is referenced from previous report (Table 1 in ref. 16). The dataset has originally 32 records, but for the 13 records in the original dataset, the proton carrier concentrations are smaller than 1 mol% because the proton carrier in those 13 glasses are originated from the residual water. Therefore, we used a dataset that consists of remaining 19 records as summarized in Table 1. Each record contains glass composition in mol% and experimentally determined *μ*_H_ at *T*_g_ and *T*_g_.

**Table 1:** Training dataset of the relationship between the glass compositions and the proton mobility (*μ*_H_) at the glass transition temperature (*T*_g_) and *T*_g_

No.	Mol% of component oxide														*μ*_H_ at *T*_g_ (cm^2^ V^−1^ s^−1^)	*T*_g_ (°C)
	HO_1/2_	NaO_1/2_	WO_3_	NbO_5/2_	TaO_5/2_	MgO	BaO	LaO_3/2_	AlO_3/2_	YO_3/2_	GdO_3/2_	GeO_2_	BO_3/2_	PO_5/2_		
1	25	3	1	8	0	0	0	5	0	0	0	0	0	58	2.1 × 10^−9^	200
2	24	8	1	8	0	0	0	5	0	0	0	0	0	54	5.5 × 10^−9^	177
3	25	10	1	8	0	0	0	5	0	0	0	0	0	51	3.7 × 10^−8^	190
4	32	6	1	8	0	0	0	5	0	0	0	0	0	48	3.7 × 10^−8^	170
5	32	8	1	8	0	0	0	5	0	0	0	0	0	46	1.2 × 10^−8^	167
6	28	2	1	8	0	0	0	5	3	3	0	0	0	50	2.0 × 10^−8^	281
7	29	6	1	8	0	0	0	5	3	0	0	0	0	48	7.6 × 10^−9^	224
8	30	5	1	8	0	0	0	5	0	3	0	0	0	48	4.1 × 10^−9^	228
9	35	0	0	3	0	5	0	3	0	0	0	2	2	50	1.3 × 10^−8^	192
10	32	3	0	3	0	0	5	3	0	0	0	2	2	50	6.8 × 10^−9^	163
11	34	2	0	4	0	0	2	4	0	0	0	4	1	49	5.4 × 10^−8^	180
12	38	2	0	0	4	2	0	4	0	0	0	2	1	47	2.7 × 10^−8^	165
13	17	8	0	0	0	0	0	8	0	0	0	1	0	66	2.6 × 10^−9^	227
14	12	13	0	0	0	0	0	6	0	0	0	6	0	63	1.3 × 10^−8^	243
15	33	2	0	0	0	2	0	5	0	0	0	5	0	53	4.0 × 10^−8^	182
16	31	4	0	0	0	2	0	0	0	0	5	5	0	53	1.2 × 10^−8^	178
17	20	5	0	0	0	0	0	6	0	0	0	6	0	63	1.5 × 10^−8^	252
18	28	7	0	0	0	2	0	0	0	0	5	5	0	53	1.4 × 10^−8^	233
19	34	1	8	8	0	0	0	5	0	0	0	0	0	44	1.1 × 10^−7^	231

### Regression models and method

A linear combination model, in which mol% of respective oxides are used as predictors, is employed for both log(*μ*_H_ at *T*_g_) and *T*_g_ in this study. The regression algorithm used in this study is based on the linear regression as implemented in MATLAB (MathWorks, USA). When the general linear regression was preliminary performed for log(*μ*_H_ at *T*_g_), the overtraining occurred maybe because of small number of training data; the predicted *μ*_H_ at *T*_g_ for the 55 296 glass compositions described later was unreasonable values in the range of 10^−29^ to 10^17^ cm^2^ V^−1^ s^−1^ (Fig. S1 and S2 in ESI†), although the range of the experimentally observed values is in the range of 2 × 10^−9^ to 2 × 10^−7^ cm^2^ V^−1^ s^−1^.^22^ Therefore, we employed the principal components analysis to fit a linear regression in order to avoid overtraining. Five principal components were employed to explain 95% of variance of original data. The mathematical model can be written as1
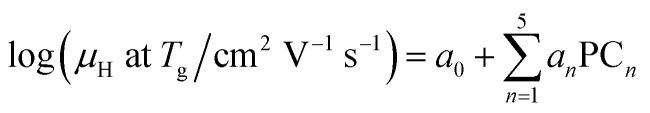
2
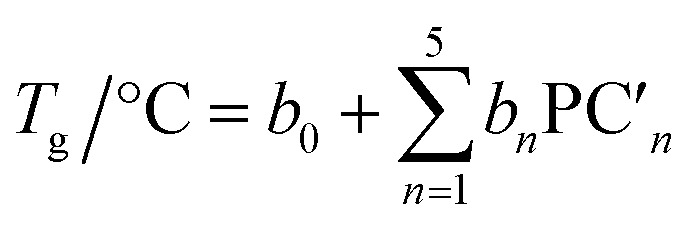
3
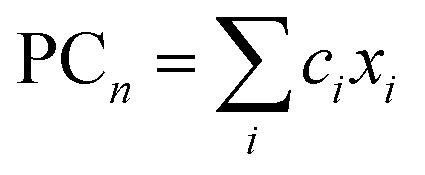
where PC_*n*_ and 
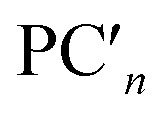
 are *n*th principal component explaining the variance of experimentally observed log(*μ*_H_ at *T*_g_) and *T*_g_, respectively, *a*_0_ and *b*_0_ are intercepts, *a*_*n*_ and *b*_*n*_ are coefficients of *n*th principal component, *x*_*i*_ is the mol% of the oxide *i*, and *c*_*i*_ is its coefficient.

In order to check the validity of the models and to understand the effect of respective component oxides on *μ*_H_ at *T*_g_ and *T*_g_, we performed to predict *μ*_H_ at *T*_g_ and *T*_g_ for 55 296 glass compositions containing 30, 33 and 36 mol% of HO_1/2_, 0, 2 and 4 mol% of WO_3_, 0, 2, 4 and 6 mol% of NbO_5/2_, 0, 2, 4 and 6 mol% of MgO, 0, 2, 4 and 6 mol% of BaO, 0, 2, 4 and 6 mol% of LaO_3/2_, 0, 1, 2, 3, 4 and 5 mol% of GeO_2_, 0, 1, 2 and 3 mol% of BO_3/2_ and 28–70 mol% of PO_5/2_. In this prediction, all the compositions were assumed to form homogeneous glasses.

## Results and discussion

### Linear regression models for *μ*_H_ at *T*_g_ and *T*_g_

The following relationships of log(*μ*_H_ at *T*_g_) and *T*_g_ against the five principal components of glass composition were obtained after regression:

4 log(*μ*_H_ at *T*_g_) = −7.8549 + 0.022233 × PC_1_ − 0.01167 × PC_2_ + 0.26874 × PC_3_ − 0.01727 × PC_4_ + 0.160456 × PC_5_,

5
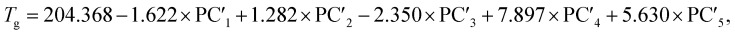


**Fig. 1:**
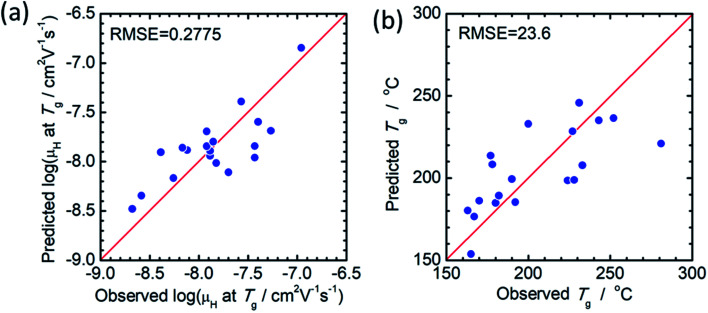
Comparison of experimentally observed and predicted values of (a) *μ*_H_ at *T*_g_ and (b) *T*_g_.

**Fig. 2:**
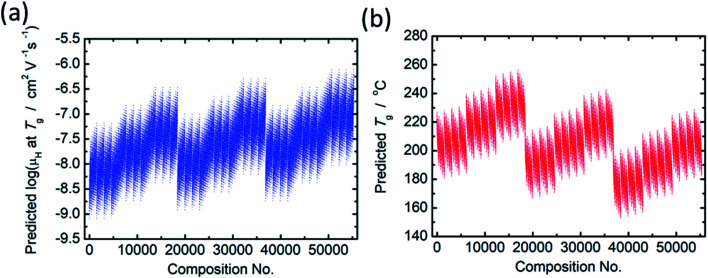
Predicted values of (a) log(*μ*_H_ at *T*_g_) and (b) *T*_g_ for the 55 296 phosphate glass compositions.

The principal components are summarized in Tables 2 and 3 for log(*μ*_H_ at *T*_g_) and *T*_g_, respectively. Fig. 1(a) and (b) show comparison of experimentally observed and predicted values of *μ*_H_ at *T*_g_ and *T*_g_, respectively, for the 19 training data. The root mean square error (RMSE) was 0.2775 for log(*μ*_H_ at *T*_g_) and was 23.6 °C for *T*_g_. No systematic error was observed and the fitting were reasonably good for both log(*μ*_H_ at *T*_g_) and *T*_g_. Fig. 2(a) and (b) respectively show the predicted values of log(*μ*_H_ at *T*_g_) and *T*_g_ for the 55 296 phosphate glass compositions. The predicted values are ranging between 8.1 × 10^−10^ and 7.7 × 10^−7^ cm^2^ V^−1^ s^−1^ for *μ*_H_ at *T*_g_ and between 152 and 256 °C for *T*_g_. As compared with experimentally determined *μ*_H_ at *T*_g_,^22^ the range of the predicted values are very close to the range of the experimentally observed values from 2 × 10^−9^ to 2 × 10^−7^ cm^2^ V^−1^ s^−1^. These results indicate that the models obtained are quite reasonable and available to discuss the effects of respective component oxides on *μ*_H_ at *T*_g_.

**Table 2:** Five principal components obtained from the analysis of *μ*_H_ at *T*_g_

Principal components		PC_1_	PC_2_	PC_3_	PC_4_	PC_5_
Proportion of variance		0.659	0.183	0.061	0.026	0.021
Cumulative proportion		0.659	0.842	0.903	0.929	0.950
Factor loading	*x*(HO_1/2_)	0.69239	−0.32693	−0.07750	−0.19571	−0.15439
	*x*(NaO_1/2_)	−0.24549	0.28854	0.71623	−0.35865	−0.03489
	*x*(WO_3_)	0.06837	0.15986	−0.11444	0.32222	0.77387
	*x*(NbO_5/2_)	0.16670	0.68417	−0.16598	0.28247	−0.30664
	*x*(TaO_5/2_)	0.02694	−0.05991	0.00355	−0.24336	0.04874
	*x*(MgO)	0.03954	−0.18457	0.01768	0.06160	−0.13778
	*x*(BaO)	0.02319	−0.04547	−0.02005	−0.12335	−0.09325
	*x*(LaO_3/2_)	−0.08309	0.19943	−0.33952	−0.50726	0.26128
	*x*(AlO_3/2_)	0.01281	0.06277	−0.04134	0.08739	−0.09090
	*x*(YO_3/2_)	0.01467	0.05828	−0.05950	0.09692	−0.09979
	*x*(GdO_3/2_)	−0.00319	−0.15212	0.31784	0.52266	−0.11954
	*x*(GeO_2_)	−0.10164	−0.37370	0.21746	0.08717	0.28694
	*x*(BO_3/2_)	0.02654	−0.05917	−0.04343	−0.10435	−0.08555
	*x*(PO_5/2_)	−0.63774	−0.25119	−0.41100	0.07224	−0.24811

**Table 3:** Five principal components obtained from the analysis of *T*_g_

Principal components		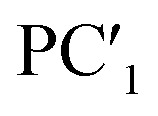	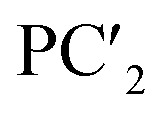	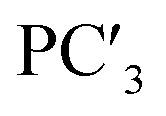	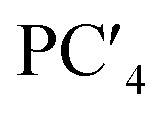	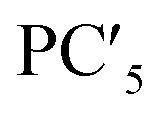
Proportion of variance		0.659	0.183	0.061	0.026	0.021
Cumulative proportion		0.659	0.842	0.903	0.929	0.950
Factor loading	*x*(HO_1/2_)	0.69239	−0.32693	−0.0775	−0.19571	−0.15439
	*x*(NaO_1/2_)	−0.24549	0.28854	0.71623	−0.35865	−0.03489
	*x*(WO_3_)	0.06837	0.15986	−0.11444	0.32222	0.77387
	*x*(NbO_5/2_)	0.1667	0.68417	−0.16598	0.28247	−0.30664
	*x*(TaO_5/2_)	0.02694	−0.05991	0.00355	−0.24336	0.04874
	*x*(MgO)	0.03954	−0.18457	0.01768	0.0616	−0.13778
	*x*(BaO)	0.02319	−0.04547	−0.02005	−0.12335	−0.09325
	*x*(LaO_3/2_)	−0.08309	0.19943	−0.33952	−0.50726	0.26128
	*x*(AlO_3/2_)	0.01281	0.06277	−0.04134	0.08739	−0.0909
	*x*(YO_3/2_)	0.01467	0.05828	−0.0595	0.09692	−0.09979
	*x*(GdO_3/2_)	−0.00319	−0.15212	0.31784	0.52266	−0.11954
	*x*(GeO_2_)	−0.10164	−0.3737	0.21746	0.08717	0.28694
	*x*(BO_3/2_)	0.02654	−0.05917	−0.04343	−0.10435	−0.08555
	*x*(PO_5/2_)	−0.63774	−0.25119	−0.411	0.07224	−0.24811

As seen in Table 2, absolute values of the factor loading of HO_1/2_ and PO_5/2_ components are particularly larger than those of the other components, indicating that *μ*_H_ at *T*_g_ is first determined by the concentration of HO_1/2_ and PO_5/2_. Taking into account that the coefficient of PC_1_ in eqn (4) is positive, *μ*_H_ at *T*_g_ increases with the increasing HO_1/2_ concentration, and it reduces with the increasing PO_5/2_ concentration. In this respect, the experimental observation that the *μ*_H_ increases with the decreasing polymerization level of phosphate glass-network is reproduced well by the present model. *μ*_H_ turns into decrease at O/P ratio (ratio of the number of oxygen to phosphorous atoms) higher than 3.5–3.6;^15^ however, such a behavior cannot be reproduced using linear regression model. Consequently, applicable composition range of the present model is limited in a O/P ratio smaller than 3.5–3.6.

**Fig. 3:**
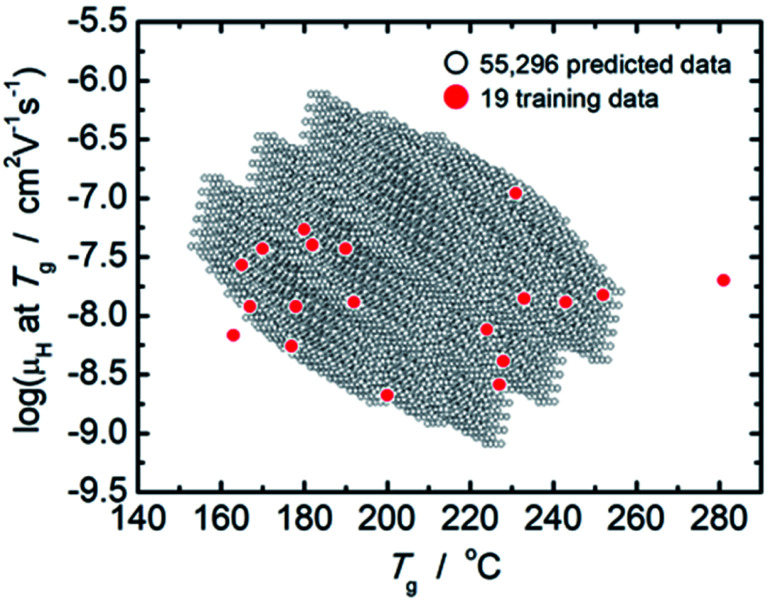
Plot of log(*μ*_H_ at *T*_g_) as a function of *T*_g_ of 55 296 predicted values (open black dots) together with the experimentally observed 19 values (closed red dots).

From comparison of the models of *μ*_H_ at *T*_g_ and *T*_g_ as summarized in Tables 2 and 3, the factor loadings of respective principal components for log(*μ*_H_ at *T*_g_) and *T*_g_ are surprisingly found to be the same each other, *i.e.*, the variance in both log(*μ*_H_ at *T*_g_) and *T*_g_ are explained by the same principal components, clearly indicating that there should be some kind of relationship between log(*μ*_H_ at *T*_g_) and *T*_g_. This is quite consistent with our previously reported estimation that the motion of protons (proton diffusion or mobility) determines the motion of glass framework (*T*_g_) in the proton conducting phosphate glasses.^22^Fig. 3 shows log(*μ*_H_ at *T*_g_) as a function of *T*_g_ of 55 296 predicted values (black dots) together with the experimentally observed 19 values (red dots). A trend that log(*μ*_H_ at *T*_g_) decreases linearly with the increasing *T*_g_ was clearly observed for the predicted values in Fig. 3. The observed relationship between log(*μ*_H_ at *T*_g_) and *T*_g_ may be a key to understand physical factor to determine *μ*_H_ at *T*_g_; however, we need additional information in order to go further this problem. Therefore, the origin of the relationship between log(*μ*_H_ at *T*_g_) and *T*_g_ remains as an open question, and we do not discuss further in this paper.

### Effects of respective component oxides on *μ*_H_ at *T*_g_ and *T*_g_

**Fig. 4:**
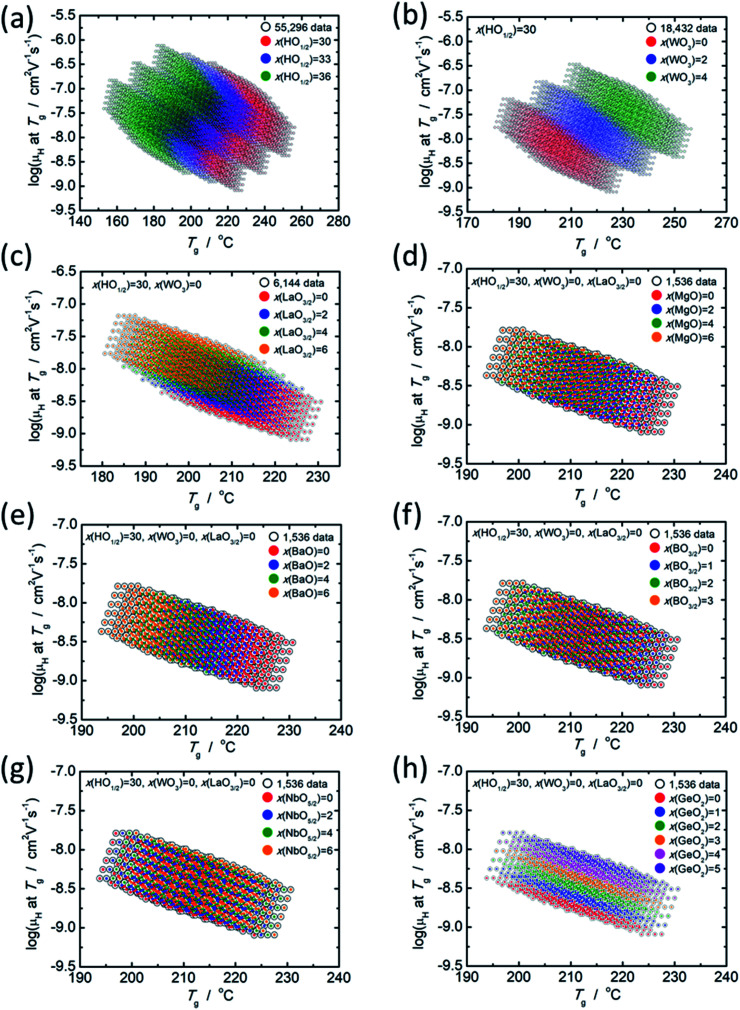
Distribution of the relationship between predicted values of log(*μ*_H_ at *T*_g_) and *T*_g_ depending on the concentration of respective component oxides. (a) 55 296 predicted values distinguished by the HO_1/2_ concentration (red dots = 30 mol% HO_1/2_, blue dots = 33 mol% HO_1/2_ and green dots = 36 mol% HO_1/2_), (b) 18 432 predicted values for the glasses with 30 mol% of HO_1/2_ distinguished by the WO_3_ concentration (red dots = 0 mol% WO_3_, blue dots = 2 mol% WO_3_ and green dots = 4 mol% WO_3_), (c) 6144 predicted values for the glasses with 30 mol% of HO_1/2_ and 0 mol% of WO_3_ distinguished by the LaO_3/2_ concentration (red dots = 0 mol% LaO_3/2_, blue dots = 2 mol% LaO_3/2_, green dots = 4 mol% LaO_3/2_ and orange dots = 6 mol% LaO_3/2_). (d), (e), (f), (g) and (h) 1536 predicted values for the glasses with 30 mol% of HO_1/2_, 0 mol% of WO_3_ and 0 mol% of LaO_3/2_ respectively distinguished by the concentration of MgO, BaO, BO_3/2_, NbO_5/2_ and GeO_2_.

As mentioned in the previous section, there is a clear relationship between log(*μ*_H_ at *T*_g_) and *T*_g_; therefore, the effect of each component oxide was studied in this regard. Fig. 4 shows the distribution of relationship between log(*μ*_H_ at *T*_g_) and *T*_g_ depending on the concentration of respective component oxides. All data plotted in Fig. 4 are predicted values. In Fig. 4(a), 55 296 predicted values are distinguished into three parts depending on the concentration of HO_1/2_. In Fig. 4(b), 18 432 predicted values for the glasses with 30 mol% of HO_1/2_ are plotted and they are distinguished into three parts depending on the concentration of WO_3_. In Fig. 4(c), 6144 predicted values for the glasses with 30 mol% of HO_1/2_ and 0 mol% of WO_3_ are plotted and they are distinguished into four parts depending on the concentration of LaO_3/2_. In Fig. 4(d), (e), (f), (g) and (h), 1536 predicted values for the glasses with 30 mol% of HO_1/2_, 0 mol% of WO_3_ and 0 mol% of LaO_3/2_ are plotted and they are distinguished into four or six parts depending on the concentration of the oxide of interest (MgO, BaO, NbO_5/2_, BO_3/2_ and GeO_2_). The situation observed, when the component oxide of interest adds into the glass as alternative to PO_5/2_, is described as follows.

With the increasing HO_1/2_ concentration (Fig. 4(a)), the *T*_g_ decreases by 5 °C per 1 mol% HO_1/2_ and log(*μ*_H_ at *T*_g_) increases by 0.06 per 1 mol% of HO_1/2_. In contrast to the dependence of HO_1/2_ concentration, both *T*_g_ and log(*μ*_H_ at *T*_g_) increases with the increasing WO_3_ concentration by 6.5 °C and 0.08 per 1 mol% of WO_3_, respectively (Fig. 4(b)). In the case of LaO_3/2_ shown in Fig. 4(c), *T*_g_ decreases with the increasing LaO_3/2_ concentration by 2.2 °C per 1 mol% of LaO_3/2_, and log(*μ*_H_ at *T*_g_) increases with the increasing LaO_3/2_ concentration by 0.1 per 1 mol% of LaO_3/2_. In the cases for MgO, BaO and BO_3/2_ shown in Fig. 4(d), (e) and (f), respectively, the dependence are similar to the case of HO_1/2_ and LaO_3/2_; *T*_g_ decreases and log(*μ*_H_ at *T*_g_) increases with the increasing concentration of the additional oxide. The variation in *T*_g_ and log(*μ*_H_ at *T*_g_) are respectively −1.5 °C and 0.05 per 1 mol% of MgO, −2.4 °C and 0.05 per 1 mol% of BaO and −2.2 °C and 0.05 per 1 mol% of BO_3/2_. For NbO_5/2_, as clearly seen in Fig. 4(g), the relationship between log(*μ*_H_ at *T*_g_) and *T*_g_ is little dependent on the NbO_5/2_ concentration, *i.e.*, *T*_g_ increases by 0.7 °C per 1 mol% of NbO_5/2_ and log(*μ*_H_ at *T*_g_) does not change regardless NbO_5/2_ concentration. In the case of GeO_2_ shown in Fig. 4(h), both *T*_g_ and log(*μ*_H_ at *T*_g_) increases with the increasing GeO_2_ concentration similar to the case of WO_3_; however, increase in *T*_g_, 0.6 °C per 1 mol% of GeO_2_, is much smaller than that of WO_3_ (6.5 °C per 1 mol% of WO_3_), while increase in log(*μ*_H_ at *T*_g_), 0.12 per 1 mol% of GeO_2_, is slightly larger than that of WO_3_ (0.08 per 1 mol% of WO_3_). These situations are summarized in Table 4.

**Table 4:** Variation of log(*μ*_H_ at *T*_g_) and *T*_g_ with the increasing component oxide by 1 mol%

Component oxide		Group 1				Group 2		Group 3
		MgO	BaO	LaO_3/2_	BO_3/2_	WO_3_	GeO_2_	NbO_5/2_
Variation per 1 mol% of oxide	log(*μ*_H_ at *T*_g_)	0.05	0.05	0.10	0.05	0.08	0.12	0.00
	*T*_g_/°C	−1.5	−2.4	−2.2	−2.2	6.5	0.6	0.7

It is noticed that the component oxides are categorized into three groups in terms of the effect on the *μ*_H_ at *T*_g_ and *T*_g_. The group 1 consists of HO_1/2_, MgO, BaO, LaO_3/2_ and BO_3/2_. They increase *μ*_H_ at *T*_g_ but decrease *T*_g_, when their concentrations increase. The group 2 involves WO_3_ and GeO_2_ that increase both *μ*_H_ at *T*_g_ and *T*_g_, when their concentrations increase. The group 3 consists of NbO_5/2_ only in the present study, and it increases *T*_g_ but does not changes *μ*_H_ at *T*_g_, when its concentration increases. Such effects categorized into three groups could not be found in the experimentally observed data, *i.e.*, 19 glass compositions that used as training data in this study. The information of the three groups is useful to obtain purpose-designed glasses.

The effect on *T*_g_ of respective group oxides is quite reasonable and explained according to the glass structural chemistry as following. The group 1 consists of the glass-modifiers except for BO_3/2_; therefore, the reduction of *T*_g_ with the increasing concentration of the group 1 oxide is reasonably understood as a result of breaking of the phosphate glass-network by introduction of the glass-modifier oxides. BO_3/2_ is a glass-former oxide, and it may exist in the glass as the trigonal planer BO_3_ in addition to the BO_4_ tetrahedron in the phosphate glasses assumed in the present study.^27–29^ When the trigonal planer BO_3_ is introduced into the glass as alternative to PO_4_ tetrahedra, the number of the bridging oxygens in the glass-network reduces as the concentration of the trigonal planer BO_3_ increases. Consequently, BO_3/2_ acts as almost glass-modifier, and its effect on *T*_g_ is similar to the other group 1 oxides that are glass-modifier oxides. The groups 2 and 3 consist of the oxides exhibiting high glass forming ability, *i.e.*, GeO_2_ is a glass-former and WO_3_ and NbO_5/2_ are conditional glass-formers.^30^ When the groups 2 and 3 oxides are introduced into the glass as alternative to PO_5/2_, GeO_2_ tetrahedra and WO_6_ and NbO_6_ octahedra strengthen the phosphate glass-network, resulting in increasing *T*_g_.

In contrast to the effect on *T*_g_, the origin of the effect on *μ*_H_ at *T*_g_ is still an open question as already mentioned. However, the effect of the group 2 oxides, *i.e.*, they increase *μ*_H_ at *T*_g_ with the increasing their concentration, may be explained phenomenologically as following. For the effect of WO_3_, we refer to the heteropoly acid of WO_3_ combined with PO_5/2_. It is well known that WO_3_ and PO_5/2_ form heteropoly acid, H_3_PW_12_O_40_·6H_2_O, and it exhibits strong acidity much stronger than H_2_SO_4_.^31,32^ The strong acidity, *i.e.*, easy proton formation, is explained by dispersion of the negative charge over many atoms of the polyanion, PW_12_O_40_^3−^, and the polarization of the outer W=O bond.^32^ Of course, the molar ratio of WO_3_ over against PO_5/2_ is much smaller (4 mol% of WO_3_ is the highest, while 28 mol% of PO_5/2_ is the lowest) than that of PW_12_O_40_^3−^; therefore, formation of PW_12_O_40_^3−^-like polyanion should be excluded. However, the WO_3_ coexisting with PO_5/2_ may have an effect to enhance acidity of ≡P–O–H units. In this case, protons are easy to dissociate from ≡P–O–H units; as a result, *μ*_H_ would be increased by the addition of WO_3_ into phosphate glasses.

In the case of GeO_2_, we refer to the silicophosphate gel that is prepared by reacting SiCl_4_ with anhydrous phosphoric acid (H_3_PO_4_).^33^ The silicophosphate gel that involves Si–O–P bondings exhibits evidently higher proton conductivity than H_3_PO_4_,^33,34^ although the increase in conductivity is not so large. Taking into account that the polymerization occurs in silicophosphate gel, the concentration of proton carriers in silicophosphate is smaller than that in phosphoric acid, indicating that the SiO_2_ addition enhances *μ*_H_. Although the reason why SiO_2_ addition enhance proton conductivity has not been fully understood yet, the octahedrally coordinated SiO_6_ that appears in silicophosphate gel is pointed out as a key feature to explain the effect of SiO_2_ addition into phosphoric acid.^33^ While GeO_2_ exhibits similar feature to SiO_2_, *i.e.*, both GeO_2_ and SiO_2_ are group 4 oxides and exhibit as glass-formers, preference of six-fold coordination of Ge^4+^ ion is higher than Si^4+^ ion. These imply that GeO_2_ would enhance *μ*_H_, when it is added into the phosphoric acid. In this case, increase in *μ*_H_ by the addition of GeO_2_ to phosphate glass would be understood by the analogous to silicophosphate gel.

## Conclusion

In summary, we developed a linear regression models for the compositional dependence of log(*μ*_H_ at *T*_g_) and *T*_g_ for the proton conducting phosphate glass based on the approach of principal component analysis, and *μ*_H_ at *T*_g_ and *T*_g_ were predicted for 55 296 of phosphate glasses involving 9 component oxide of HO_1/2_, MgO, BaO, LaO_3/2_, WO_3_, NbO_5/2_, BO_3/2_, GeO_2_ and PO_5/2_. The models themselves do not have any physical meaning of course, but they provide the following information about the effects of respective component oxides on *μ*_H_ at *T*_g_ and *T*_g_: (i) the *μ*_H_ at *T*_g_ is determined first by concentrations of HO_1/2_ and PO_5/2_; *μ*_H_ at *T*_g_ increases with increasing HO_1/2_ concentration and decreasing PO_5/2_. (ii) There is a trend for log(*μ*_H_ at *T*_g_) to increase linearly as *T*_g_ decreases. This is quite consistent with our estimation previously reported that the motion of protons determines the motion of glass framework in the proton conducting phosphate glasses. (iii) The component oxides are categorized into three groups according to the effects on *μ*_H_ at *T*_g_ and *T*_g_. The group 1 oxides that behave as glass-modifiers increase *μ*_H_ at *T*_g_ and decrease *T*_g_, and HO_1/2_, MgO, BaO and LaO_3/2_ and BO_3/2_ are involved in this group. The group 2 oxides increase both *μ*_H_ at *T*_g_ and *T*_g_, and WO_3_ and GeO_2_ are involved in this group. The group 3 oxides increase *T*_g_ but do not vary *μ*_H_ at *T*_g_. Only NbO_5/2_ falls into the group 3 among the oxides examined in this study. These information are very useful to obtain purpose-designed glasses; therefore, they will be applied to the future development of proton-conducting phosphate glasses. Especially, the effects of the additional glass-formers, such as GeO_2_ and WO_3_, are very important to design highly proton conducting phosphate glass at intermediate temperatures.

The enhance of *μ*_H_ at *T*_g_ by WO_3_ and GeO_2_ of group 2 oxide is phenomenologically understood by referring to the strong acidity of PW_12_O_40_^3−^ heteropoly acid and the enhancing *μ*_H_ of phosphoric acid by SiO_2_ addition, respectively. In contrast, the origin of the effect of groups 1 and 3 oxides on *μ*_H_ at *T*_g_ and the relationship between log(*μ*_H_ at *T*_g_) and *T*_g_ still remain as open questions.

## Author contributions

Takahisa Omata: conceptualization, methodology, formal analysis, software, writing – original draft and visualization, Issei Suzuki: validation, visualization and writing – review & editing, Aman Sharma: formal analysis and data curation, Tomohiro Ishiyama: writing – review & editing, Junji Nishii: conceptualization, funding acquisition and writing – review & editing, Toshiharu Yamashita: supervision and writing – review & editing, Hiroshi Kawazoe: supervision and writing – review & editing.

## Conflicts of interest

The authors declare no competing interests.

## Supplementary Material

RA-011-D0RA10327F-s001
