# Supplementary material for: Understanding the effect of oxide components on proton mobility in phosphate glasses using a statical analysis approach
Source: RSC Adv. 2021 Jan 14;11(5):3012–9. doi: 10.1039/d0ra10327f (PMC8693833; doi:10.1039/d0ra10327f)
Supplement: RA-011-D0RA10327F-s001 [file RA-011-D0RA10327F-s001.pdf]

Electronic Supplementary Material (ESI) for RSC Advances.
This journal is © The Royal Society of Chemistry 2021

Supplementary Information

Understanding the effect of oxide components on proton mobility in phosphate glasses using a statical analysis approach

Takahisa Omata,[*a] Issei Suzuki,[a] Aman Sharma,[a] Tomohiro Ishiyama,[b] Junji Nishii,[c] Toshiharu Yamashita[d] and Hiroshi Kawazoe[d]*

[a] *Institute of Multidisciplinary Research for Advanced Materials, Tohoku University, Katahira 2-1-1, Sendai 980-8577, Japan*
[b] *Fuel Cell Materials Group, Research Institute for Energy Conservation, National Institute of Advanced Industrial Science and Technology (AIST), AIST Central 5, Higashi 1-1-1, Tsukuba, Ibaraki 305-8565, Japan*
[c] *Research Institute for Electronic Science, Hokkaido University, Kita 21 Nishi 10, Kita-ku, Sapporo 001-0021, Japan*
[d] *Kawazoe Frontier Technologies Corporation, Kuden-cho 931-113, Sakae-ku, Yokohama 247-0014, Japan*

S1. Linear Regression models

We first employed a general linear combination model, in which mol% of respective oxides are used as predictors, for both log(μ_H at T_g) and T_g. The regression algorithm used in this study is based on the linear regression as implemented in MATLAB (MathWorks, USA). The obtained model for log(μ_H at T_g) is as following:

$$\begin{aligned}\log(\mu H \text{ at } Tg) = &-0.236388563x(HO_{1/2})-0.21732199x(NaO_{1/2})-0.128502192x(WO_3)\\ &+0.671171102x(NbO_{5/2})+1.029198044x(TaO_{5/2})+1.374093231x(MgO)\\ &+1.306366399x(BaO)+1.971135165x(LaO_{3/2})-0.201698121x(AlO_{3/2})\\ &-0.284685842x(YO_{3/2})+1.859907493x(GdO_{3/2})+0.754725434x(GeO_2)\\ &-0.629475199x(BO_{3/2})-0.293206847x(PO_{5/2}) \qquad (S1)\end{aligned}$$

Figure S1 shows comparison of experimentally observed and predicted values of μ_H at T_g. The root mean square error (RMSE) was 0.2278 and reasonably small. However, as shown in Fig. S2, the predicted values of log(μ_H at T_g) for the 55,296 phosphate glass compositions (see the main text) were unreasonable in the range of 10^{-29}~10^{17} cm^{2}V^{-1}s^{-1}, although the range of the experimentally observed values is 2×10^{-9}~2×10^{-7} cm^{2}V^{-1}s^{-1}. This indicates that the overtraining occurred maybe because of small number of training data.

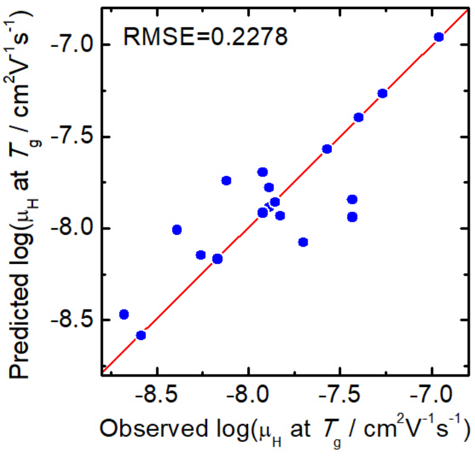

Figure S1. Comparison of experimentally observed and predicted values of μ_H at T_g

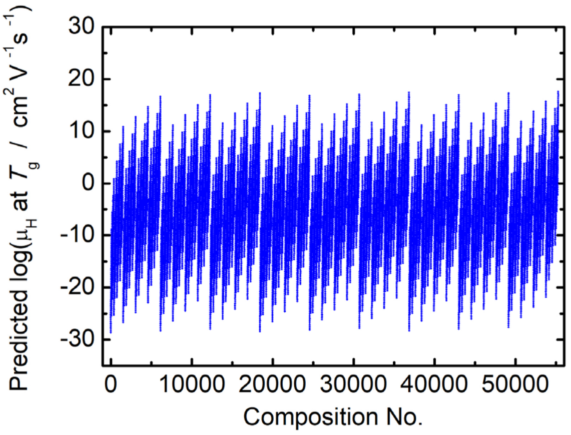

Figure S2. Predicted values of log(μ_H at T_g) for the 55,296 phosphate glass compositions.
